# Analysing Remote Nocturnal Activity and Sleep Monitoring Data to Develop Predictive Models in Dementia Care

**DOI:** 10.1002/alz.087967

**Published:** 2025-01-09

**Authors:** Payam Barnaghi

**Affiliations:** ^1^ Imperial College London, London, United Kingdom; UK Dementia Research Institute, Care Research and Technology Centre, London United Kingdom

## Abstract

**Background:**

Close to 23% of unplanned hospital admissions for people living with dementia (PLWD) are due to potentially preventable causes such as severe urinary tract infections (UTIs), falls, and respiratory problems. These affect the well‐being of PLWD, cause stress to carers and increase pressure on healthcare services.

**Method:**

We use routinely collected in‐home sensory data to monitor nocturnal activity and sleep data.

**Result:**

We apply machine learning models to develop a new generation of dementia care solutions to analyse changes in patterns.

**Conclusion:**

Developing machine learning models to analyze in‐home sleep data will help enable detection of sleep changes and timely interventions.

